# Effects of a death education based on narrative pedagogy in a palliative care course among Chinese nursing students

**DOI:** 10.3389/fpubh.2023.1194460

**Published:** 2023-11-09

**Authors:** Yuanyuan Zhu, Yamei Bai, Aihong Wang, Yuexian Liu, Qinyi Gao, Zhi Zeng

**Affiliations:** ^1^School of Nursing, Nanjing University of Chinese Medicine, Nanjing, Jiangsu, China; ^2^Nursing Department, Nanjing Jiangning Hospital, Nanjing, Jiangsu, China; ^3^School of Health Economics and Management, Nanjing University of Chinese Medicine, Nanjing, Jiangsu, China

**Keywords:** death education, narrative pedagogy, palliative care, nursing, China

## Abstract

**Background:**

Death education has been confirmed to be an effective method to enhance nursing students’ attitudes and coping abilities toward death. However, integrated Narrative Pedagogy into the death education to explore educational effectiveness is still limited. The study aimed to evaluate the effects of a death education based on Narrative Pedagogy in a palliative care course on the attitude toward death, coping with death, and attitude toward caring for the dying among undergraduate nursing students in China.

**Methods:**

The study was designed as a pre-post intervention study with a quasi-experimental design. All the participants received 6 class hours of death education which was designed in a palliative care course. The death education includes preparation, presentation, discussion, reflection, and practice of the narrative materials. Death Attitude Profile-Revised (DAP-R), Coping with Death Scale (CDS), and Frommelt Attitude Toward Care of the Dying-Form B (FATCOD-Form B) were used to measure outcomes.

**Results:**

Sixty undergraduate nursing students who consented. There were statistically significant increases in the mean score of neutral acceptance and approach acceptance in the DAP-R, CDS, and FATCOD-Form B before and after the intervention.

**Conclusion:**

This death education integrated Narrative Pedagogy which indicated to improve attitude toward death, competence to cope with death, and attitude toward the care of dying patients. The findings assist teachers in understanding the importance and urgency of death education, as well as providing a favorable approach to death education. However, the long-term effectiveness still needs to study in further.

## 1. Introduction

According to the definition of the World Health Organization, palliative care is “an approach that improves the quality of life of patients (adults and children) and their families who are facing problems associated with a life-threatening illness. It prevents and relieves suffering through the early identification, correct assessment and treatment of pain and other problems, whether physical, psychosocial or spiritual” (1). Each year, there are 40 million people in need of palliative care worldwide, accounting for 45.3% of all deaths in 2017. However, only 14% of people who need palliative care receive it. China’s population mortality rate in 2021 is 7.2 per 1,000 (2). Chronic diseases account for 88.5% of total deaths in China in 2019 (3). Palliative care in China is in the midst of development, with more than 1,000 nurses working in the field (4). Along with the increase of the aging population and the burden of diseases, the need for palliative care will continue to grow, but the health workforce is insufficient to meet its development (1, 5). Nurses are usually the care providers who accompany the patients and their families the most of time during palliative care, together with the specificity of the patients, nurses are often faced with death and dying. Death is a complex problem in life that everyone has to face. When facing death, there are complex changes in people’s psychology and behavior. According to the Terror Management Theory (TMT), on the one hand, humans possess a sense that death is inevitable. On the other hand, humans have the instinct of self-preservation, and the conflict between them leads to death anxiety and a lower sense of well-being. Thus, people respond to this threat through various psychological buffer mechanisms (6). Despite people having buffer mechanisms, nursing students and new graduate nurses still reported negative feeling experiences during taking care of dying patients, such as nervousness, helplessness, anxiety, uncertainty, and frustration (7, 8). Although nurses believed it’s important to support the patient and facilitate a good death, they found it inadequate to accept patients’ imminent death (9, 10). Nurses’ personal views and attitudes toward death were also related to attitudes toward palliative care (11). Thus, comprehensive death education is required to improve nursing students’ attitudes toward death and palliative care.

Death is a normal phenomenon and a natural process in the life course. However, death is considered to be taboo and avoided to be talked about due to the influence of traditional Chinese culture. This situation makes it difficult for nurses to improve their attitude toward death. Meanwhile, in mainland China, there is a huge need for death education among nurses and nursing students (12). This contradictory status makes the implementation of death education more urgent. Death education originated in the United States and then developed in the United Kingdom, Japan, Germany, and other countries rapidly. Compared with Hong Kong and Taiwan, the development of death education in mainland China has been lagged due to the influence of traditional culture (12, 13). As of 2018, fewer than 20 universities in mainland China have set up death education courses for students (14). Death education aims to promote the development of students’ emotions, attitudes, and literacy as well as to enable them to master professional skills (15). Death education has been proved to be an effective approach to improving attitude toward death and work efficiency (15–17).

Death is a sensitive topic, so it’s important to choose an appropriate education approach. The most common form of death education is the lecture, and an American study showed that a one-hour lecture was able to motivate nursing students to give more consideration to the needs of patients in the clinical work (18). Nevertheless, conventional pedagogy has its limitations which include a lack of flexibility, experience, and interactivity. Narrative Pedagogy can be used to investigate topics that are difficult to talk about (19). Narrative Pedagogy is a phenomenological pedagogy that refers to the achievement of educational and research goals via the narrative, explanation, and reconstruction of stories and experiences. The first application of Narrative Pedagogy in the field of nursing occurred in the 1990s when Diekelmann adapted Narrative Pedagogy to nursing education (20). McDrury et al. proposed the Reflective Learning through Storytelling Model which includes five stages: story finding, storytelling, story expanding, story processing, and reconstructing story. This model provides teachers with a standardized teaching procedure as well as methodological guidance for using Narrative Pedagogy (21). Narrative Pedagogy requires teachers and students to work together through reflection, interpretation, and discussion of their shared experience to reach an understanding of the meaning of the story (22). The connotation of Narrative Pedagogy is constantly deepening, not only to share stories, experiences, or provide examples of specific patients, but to create a situation for nurses to reflect and improve empathy (23). There have been studies that combine Narrative Pedagogy with nursing professional education which has shown beneficial to form the professional nursing identity and stimulate creative thinking (22, 24). However, research on Narrative Pedagogy-based death education is insufficient. Given the sensitivity of the topics of death education and the advantages of Narrative Pedagogy, integrating the two will be a rewarding attempt. The purpose of the study was to evaluate the effects of a death education based on Narrative Pedagogy in a palliative care course on the attitude toward death, coping with death, and attitude toward caring for dying patients among undergraduate nursing students in China.

## 2. Methods

### 2.1. Study design

The study used a single-group design with a pre-test, post-test design. This study was conducted from October to November 2021.

### 2.2. Participants and setting

This is a major elective course. Participants were sophomore nursing students in a Chinese medical university who chose the palliative care course and were willing to participate in this research would be invited. The researcher would introduce the purpose, process, and information to be gathered in this study during the first class. Participation in this study was entirely voluntary, and whether or not a student participated did not affect the final grade. Written informed consent was obtained from each participant. The sample size was determined by G*Power. We considered an effect size of 0.61, an alpha error probability of 0.05, and a power of 0.95. According to the previous studies, we used the score of attitude toward caring for dying patients and their family members as a reference (25). The estimated sample size was 37. Finally, a total of 60 undergraduate nursing students participated in this study. The authors assert that all procedures contributing to this study comply with the Helsinki Declaration as revised in 2013 and the ethical standards of the relevant national and institutional committees on human experimentation. Research permission was obtained from the Ethics Committee of the Nanjing Jiangning Hospital (No. 2019-03-025-K01).

### 2.3. Intervention

The contents and teaching approaches of this course were developed by two clinical nursing experts and four nursing teachers. The researcher identified a preliminary proposal by reviewing the literature, sorting out the current research status in China and abroad, and holding expert symposia to meet the purposes of the study. Later, the final project was formed after several discussions. Since this course was designed for undergraduate students rather than specialist nurses, there were 18 class hours (1 class hour = 40 min) in the palliative care course, of which 6 class hours were used for death education. According to McDrury’s narrative pedagogy, the Chinese researchers combined with the narrative nursing materials to design a narrative pedagogy suitable for Chinese nursing students which consists of the following 5 steps (26). The outline of the death education was presented in Table 1.

**Table 1:** Outline of the death education.

Step	Stage	Subject	Content	Time
Step 1	Preparation	Choose and design narrative materials	Researchers choose suitable narrative materials Assign tasks before class: learn the theoretical content, preview the narrative materials, and write narrative stories	Before class
Step 2	Presentation	Present materials and create the situation	Present narrative materials: Ready-made narrative materials: two films (Departures, The Bucket List), a documentary (Life Matters), and a literary work (Being Mortal: Medicine and What Matters in the End) Self-made narrative materials: stories of students’ own experiences of life and death	1 class hour
Step 3	Discussion	Inspire discussions and experience empathy	Set up questions based on the narrative materials Discuss in 6 groups Share opinions about life and death	1 class hours
Step 4	Reflection	Guide reflections and share insights	Guide students to reflect Share new inspirations and values of death	1 class hours
Step 5	Practice	Expand connotations and strengthen practice	Students are divided into 6 groups Role-playing based on a self-created script Every student completes a reflective writing after class	3 class hours

Step 1: Choose narrative materials and design the teaching strategy.

To diversify the narrative materials, we discussed the characteristics that they should have. The narrative materials included, but were not limited to, films, documentaries, and literary works and should be educational, inspirational, and artistic. The researchers searched Chinese and foreign databases and web pages to find as many materials as possible. These collected materials were categorized and discussed in the expert symposia to select materials suitable for this study. Therefore, the narrative materials used here were partly derived from previous studies and partly from personal selection (27). Finally, the present study used classic films, literature, documentaries, and personal experiences as the main narrative materials. Before the class, the teacher provided complete narrative materials on the online teaching platform, and students would learn the theoretical content, as well as preview the narrative materials that would be used in the class. Furthermore, students were divided into six groups, with each group choosing one student to recall his or her memories about death and write narrative materials to be shared in class.

Step 2: Present materials and create the situation.

Due to the different forms of narrative materials, the way of presentation was also different. The ready-made narrative materials in this study were two films (*Departures, the Bucket List*), a documentary (*Life Matters*), and a literary work (*Being Mortal: Medicine and What Matters in the End*). Due to the limited time in class, the teacher chose some fragments of films and documentary films to play in the classroom. For example, in the *Departures*, the leading actor cared for the corpse of the dead in front of her family members. The whole process was solemn and warm so that the students could experience respect for life. In the *Being Mortal: Medicine and What Matters in the End*, the author’s views on aging, disease, death, and how to understand and make decisions about life and death were shared with students. In contrast to the materials mentioned above, the research conducted a session with students serving as narrative subjects. Students would share the self-made narrative materials in the form of compelling presentations during the class.

Step 3: Inspire discussions and experience empathy.

After the narrative materials were presented, the teacher set several questions to guide students in interpreting the story, analyzing the motivations of the character’s actions, and exploring possible solutions in groups. The problem’s setting focuses on the details, connotations, and emotions that students often overlook. For example, following a watching of a segment of The Bucket List, the following questions were presented: When Edward knew that death was imminent, why would he change his mind and attitude about treatment and then decide to realize his wishes? If you are Edward’s relative, could you understand his decision? The teacher encouraged each student to express their opinions, feelings, and allow students to discuss freely.

Step 4: Guide reflections and share insights.

The teacher guided students to reflect on their own experiences, exchange and share their views and perceptions on death and life based on the analysis and discussion of narrative materials. New inspirations and values were gradually formed during the process of expressing opinions and reflections, which fosters the generation of perceptions.

Step 5: Expand connotations and strengthen practice.

The students were divided into six groups. Each group of students completed a script on the theme of life and death based on the true stories they have experienced. The script should underline the value of human life and preserve the dignity of patients. Patients, doctors, nurses, family members, volunteers, and so on were all assigned different roles. Students act out the scripted part in class, experiencing the emotion and connotation of the role and strengthening their understanding of death. After class, each student completed reflective writing which contains understanding and perception of death, life, palliative care, and the future development of death education in China.

### 2.4. Study instruments

The study used a questionnaire packet that required demographic information such as age, gender, living area, religion, and some previous experiences. Moreover, participants responded to three structured scales which were Death Attitude Profile-Revised (DAP-R), Coping with Death Scale (CDS), and Frommelt Attitude Toward Care of the Dying-Form B (FATCOD-Form B).

DAP-R is a structured scale to measure attitudes toward death. The scale includes 32 items in 5 domains which are fear of death (7 items), death avoidance (5 items), neutral acceptance (5 items), escape acceptance (5 items), and approach acceptance (10 items). Neutral acceptance means that death is a natural part of life and is neither feared nor welcomed, which reflects a more peaceful state. Approach acceptance refers to believing that death is a gateway to a happy afterlife and that there is a happy life after death. Embracing death as a means of escaping from suffering is an example of escape acceptance. The answers are given on a 5-point Likert scale ranging from 1 (strongly disagree) to 5 (strongly agree). The scale’s overall score ranges from 32 to 160 points, and each domain’s score is the sum of item scores divided by the number of items. The higher the score, the more a person’s attitude regarding death is oriented toward this domain. Tang et al. translated the scale into Chinese in 2014. Cronbach’s α coefficient of the total scale was 0.875 and the split-half coefficient was 0.864 (28).

Bugen et al. developed and applied CDS in 1980 to assess the competence in dealing with death and their professional knowledge concerning preparedness for death (29). The Chinese version of CDS contains 28 items using a 7-point Likert scale to score from completely disagree (1 point) to completely agree (7 points). The total score of CDS ranges from 28 to 196, the higher the score representing the higher level of coping with death. Scale-level content validity index (S-CVI) was 0.987 and item-level content validity indexes (I-CVIs) were between 0.832 and 1.000. Cronbach’s α coefficient of the total scale and split-half coefficient was 0.905 and 0.784, respectively, and the test–retest reliability was 0.973. The Chinese version of CDS has good reliability and validity (30).

Frommelt et al. developed the FATCOD-Form B in 2003 to evaluate the attitude toward caring for dying patients and their family members (31). FATCOD-Form B includes 30 items containing an equal amount of positive and negative items. Positive items are scored as following: 1 = strongly disagree, 2 = disagree, 3 = uncertain, 4 = agree, 5 = strongly agree. Negative items score in reverse. Positive attitude toward caring for the dying patient and perception of patient- and family-centered care are two domains in the scale. The total score was from 30 to 150, with a higher score indicating a more positive attitude. In the Chinese version of FATCOD-Form B, Cronbach’s α coefficient of the total scale was 0.805 and the split-half coefficient was 0.697 (32).

### 2.5. Data collection and analysis

Continuous variables in the study were normally or approximately normally distributed. The results of the descriptive statistics were expressed as a number of participants, percentage, mean ± standard deviation (SD). The paired *t*-tests were used to compare the scores before and after the intervention, with a level of significance set at *p* < 0.05. In order to providing a more objective understanding of the actual degree of difference between pre- and post-intervention, effect size also been calculated by (mean_pre-intervention_-mean_post-intervention_)/standard deviation. The Statistical Package for Social Sciences (SPSS) version 22.0 was used to analyze the data (SPSS, Inc., Chicago, IL, United States).

## 3. Results

### 3.1. Characteristics of participants

The demographic characteristics of 60 participants are shown in Table 2. The mean age was 22.38 ± 0.12 years and 59 (98.3%) of them were female and unmarried. Most of the participants (63.3%) were living in the city. All the students were of Han nationality and have no religious belief. Only 9 participants participated in courses or trainings on death and dying before. 55% of participants had previous bereavement experience. Of them, 28.3% had previous experience of taking care of dying patients and 26.7% had previous experience of taking care of dying relatives or friends.

**Table 2:** Demographic characteristics (*N* = 60).

Characteristic	*N* (%) or mean ± SD
Age (years)	22.38 ± 0.12
Gender	
Male	1 (1.7)
Female	59 (98.3)
Marital status	
Married	1 (1.7)
Unmarried	59 (98.3)
Health status	
Healthy	60 (100)
Unhealthy	0 (0)
Living area	
City	38 (63.3)
Town	14 (23.3)
Rural area	8 (13.3)
Religion	
Yes	0 (0)
No	60 (100)
Nationality	
Han	60 (100)
Others	0 (0)
Participated in courses or trainings on death and dying before	
Yes	9 (15)
No	51 (85)
Previous bereavement experience	
Yes	33 (55)
No	27 (45)
Previous experience of taking care of dying patients	
Yes	17 (28.3)
No	43 (71.7)
Previous experience of taking care of dying relatives or friends	
Yes	16 (26.7)
No	44 (73.3)

### 3.2. Pre- and post-test differences in DAP-R, CDS, and FATCOD-Form B

Table 3 shows that before the intervention, the mean score of neutral acceptance was the highest, while the mean score of escape acceptance was the lowest. The mean scores of each domain, in descending order, were neutral acceptance, death avoidance, fear of death, approach acceptance, and escape acceptance. After the intervention, the mean score of neutral acceptance remained the highest, while the mean score of escape acceptance remained the lowest, and the rankings of death avoidance and fear of death decreased. There were statistically significant increases in the mean score of neutral acceptance (3.85 ± 0.41 vs. 3.98 ± 0.54, *t* = 2.021, *p* = 0.048) and approach acceptance (2.90 ± 0.58 vs. 3.04 ± 0.59, *t* = 2.039, *p* = 0.046).

**Table 3:** Scores of DAP-R, CDS, and FATCOD-Form B before and after the death education (*N* = 60).

	Pre-test	Post-test	*t*	Effect size	*p* value
**DAP-R**					
Fear of death	2.95 ± 0.71	2.85 ± 0.77	−1.530	0.198	0.131
Death avoidance	3.05 ± 0.60	2.95 ± 0.70	−1.245	0.161	0.218
Neutral acceptance	3.85 ± 0.41	3.98 ± 0.54	2.021	−0.261	0.048
Escape acceptance	2.80 ± 0.76	2.81 ± 0.82	0.065	−0.008	0.948
Approach acceptance	2.90 ± 0.58	3.04 ± 0.59	−6.727	−0.263	<0.001
**CDS**					
Total score	117.29 ± 15.97	125.45 ± 19.74	7.022	−0.396	<0.001
**FATCOD-Form B**					
Total score	100.60 ± 9.60	103.93 ± 10.26	2.694	−0.348	0.009
Positive attitude toward caring for the dying patient	45.45 ± 6.31	47.28 ± 7.16	2.161	−0.279	0.035
Perception of patient- and family-centered care	54.92 ± 5.77	56.65 ± 6.65	5.039	−0.266	<0.001

DAP-R, death attitude profile-revised; CDS, coping with death scale; FATCOD-Form B, Frommelt attitude toward care of the dying-Form B.

The mean total score of CDS was 117.29 before the intervention, a significant improvement was shown in CDS after intervention (*t* = 3.067, *p* = 0.003).

After the intervention, the mean total score of FATCOD-Form B was 103.93, which was significantly higher than 100.60 before the intervention (*t* = 2.694, *p* = 0.009). There were statistically significant differences in mean scores of a positive attitude toward caring for the dying patient (45.45 ± 6.31 vs. 47.28 ± 7.16, *t* = 2.161, *p* = 0.035) and perception of patient- and family-centered care (54.92 ± 5.77 vs. 56.65 ± 6.65, *t* = 2.059, *p* = 0.044) pre-and post-intervention.

## 4. Discussion

In the present study, only 15% of students participated in courses or trainings on death and dying before the intervention. This result was similar to a previous study that was also conducted in China (15). Although more than half of the participants reported that they had previous bereavement experience, the mean score of death avoidance was ranked relatively high before the intervention, indicating that students tend to evade thinking about death and discussing topics related to death. Under the influence of traditional Chinese culture on death, it is not encouraged to talk about death in public. People tend to avoid facing death which is related to the fear and unknown of death. The DAP-R had the highest mean score of neutral acceptance, but the mean scores of escape acceptance and approach acceptance were low, which was consistent with another Chinese study (33). However, the mean scores of escape acceptance and approach acceptance were higher than neutral acceptance among nursing students in a Turkish study which did not reflect a similar result compared with the present study (34). Due to cultural and religious differences, none of the participants in this study had a religious belief, but the majority of people in Turkey believe in Islam. Attitude toward death was related to religion which can go some ways to alleviating death anxiety and fear, then lead to different attitudes toward death (34).

After the intervention, there was an overall change in the score of 5 DAP-R domains. Notably, there were statistically significant increases in the mean score of neutral and approach acceptances, indicating that death education influence students’ attitudes toward death. Anyone would find facing death difficult. When nurses see a patient’s death or dying while at the work, it’s normal for them to feel helpless and scared. Death education differs from traditional theoretical education and is associated with positive psychology, with a greater emphasis on the importance of the unity of mind, spirit, and body (35). For nurses, death education is to cultivate nurses’ abilities so that they could face death, and contribute to changes in the perception, behavior, and attitude in the long term (36). The results of this study confirm the need for and importance of death education, as well as its effect on improving attitude toward death. Based on the TMT, researchers later combined psychodynamics and cognitive science to propose the Cognitive Dual-process Model. This model suggested two defense mechanisms against death anxiety: proximal and distal defenses, where proximal defenses are at the conscious level, and distal defenses are at the subconscious level (37). In this study, watching, reading, sharing, and thinking about the comprehensive narrative materials made students aware of the inevitability of death, a process named Mortality Salience (MT), indicating that the proximal system was used. People could use suppression or rationalization to remove the death anxiety from their consciousness. However, this fade-out is not a complete disappearance but a movement to the subconscious level. The active death thinking in the preconscious triggers the activation of anxiety-buffering mechanisms, including cultural worldview, self-esteem, and close interpersonal relationships (38). This theory might explain the fluctuation of students’ attitude toward death.

Before the intervention, the total score of CDS was 117.29, a relatively low total score than a Spanish study (39). The original CDS had 30 items, but two of them have been deleted from the Chinese version, therefore the total score of the Chinese scale will be lower than the total score of the original scale. Even taking into account the difference in total score, the participants in this study had a lower ability to cope with death. The majority of the students in the Spanish study were of Christianity and Islam. The influence of religion and culture on perception is profound and should not be ignored. Religion is not only a belief but also directs the perception of society, health, life, and death (39). The score of FATCOD at the baseline was similar to another Chinese study (40), but lower than Mastroianni et al. study (41). A higher score was significant about the experience of palliative care training and taking care of terminally ill patients (41). However, 85% in the current study had never received professional trainings on death and dying, and 71.7% have no previous experience caring for dying patients. The lack of enough nursing experience and training might explain why they have fewer positive attitudes toward the care of dying patients. According to Kesselheim et al. (42), pediatric hematology-oncology fellows have a strong need for humanism and professional education, and 85 percent of participants would want to have more opportunity to learn about death and dying. Therefore, it is essential to provide death education for nursing students and health care staff.

Innovatively, the present study developed death education using a narrative approach has shown significant results in improving the attitude and ability to cope with death, as well as attitude toward caring for dying patients. The study utilized the modified framework of McDrury’s Narrative Pedagogy which contained five steps to integrate rich narrative materials including films, documentaries, literature, personal experiences, and self-authored script, allowing students to engage in the class and promoting reflection through the sharing and practicing of narrative materials. Films are common narrative materials and are more vivid than oral presentations which make participants more immersive, attracting their attention and promoting active thinking and reflection. Head et al. integrated contemporary films into the death and grief course has shown to be effective in facilitating student discussion and reflection (43). An Italian study using psychodrama and moviemaking in death education, spirituality, and the meaning of life were improved while annihilation and alexithymia were reduced in 138 high school students in the experimental group (44). These positive results indicated that the application of visual materials such as film clips to death education has its unique advantages. Apart from films, our study indicated that allowing students to practice and play a certain role in a specific script can also increase their willingness to engage in caring for the dying. The result was consistent with a Turkish study that enrolled creative drama into education among nursing students and confirmed it to be effective in improving students’ attitudes toward caring for dying patients (45). Narrative Pedagogy does not completely subvert conventional education, but rather reinforces learning through the form of storytelling and reflection. Narrative Pedagogy, because of the variety of narrative materials, may convey not only knowledge but also values and cultures, which can have a more deep influence on students’ thinking and practices (46). Additionally, Narrative Pedagogy is a flexible educational approach in which students and teachers can learn together, attaching importance to students’ inner experience, enabling a more intimate partnership. This harmonious atmosphere facilitates interaction and discussion in class (47). Students in the current study had the opportunity to discuss and reflect on the issue of death, acquiring a better understanding of the value and importance of palliative care.

Some limitations should be mentioned. Firstly, due to the setting of the palliative care course, the number of class hours used for death education was limited. Compared to previous studies, the class hours in the present study were short (15, 48). There were only 6 class hours, and the researcher could only share minimal materials in the present death education. Additionally, the time for group discussion was restricted, and not every student had the opportunity to share their opinions. This limitation may be the reason for the small effect size. Therefore, optimizing study design may result in more significant results. Secondly, we used only a quantitative method to evaluate the efficiency of death education. Qualitative research methods should be considered to further assess students’ emotional experiences. Semi-structured interview method can be used to collect qualitative data on emotional experience (49). Thirdly, according to the principles set out in the syllabus, all the 60 students should be in one class and could not be divided into different class. We have no way to take two interventions in the same time and space, so no control group was established. If this course is expanded in the future and students can be divided into two groups, then a control group can be set up.

Despite these limitations, this study brought us meaningful results. Future studies should increase the number of class hours for death education to give more students the opportunity to express their opinions, and set up a control group to further illustrate the effects of Narrative Pedagogy in the death education. Moreover, mixed research methods could be used in future studies.

## 5. Conclusion

This death education integrated Narrative Pedagogy in a palliative care course among undergraduate nursing students, which improved the competence to cope with death and attitude toward the care of dying patients, as well as changed attitude toward death. Whether or not students are engaged in palliative care in future clinical work, being able to cope with death is a necessary capacity. The findings of this study help educators in better understanding the importance and urgency of death education, as well as providing a new approach to death education. Death education is a very important part of palliative care. Universities and health care centers in China should increase focus on death education and offer courses or training to help students and clinical nurses better cope with death and thus improve the quality of palliative care. The use of Narrative Pedagogy to death education produced significant results but effect sizes were small. Thus, further improvements of the study design and intervention are still needed in future studies.

## Data availability statement

The raw data supporting the conclusions of this article will be made available by the authors, without undue reservation.

## Ethics statement

The studies involving humans were approved by Ethics Committee of the Nanjing Jiangning Hospital. The studies were conducted in accordance with the local legislation and institutional requirements. The participants provided their written informed consent to participate in this study.

## Author contributions

YZ: conceptualization, data curation, formal analysis, investigation, methodology, software, project administration, writing – original draft, and writing – review and editing. YB: conceptualization, data curation, formal analysis, investigation, writing – original draft, and writing – review and editing. AW: conceptualization, data curation, formal analysis, and writing – original draft. YL: conceptualization, methodology, and writing – original draft. QG and ZZ: conceptualization, supervision, project administration, writing – original draft, and writing – review and editing. All authors contributed to the article and approved the submitted version.
